# The rise and fall of women’s advantage: a comparison of national trends in life expectancy at age 65 years

**DOI:** 10.1007/s10433-013-0274-8

**Published:** 2013-03-22

**Authors:** Mats Thorslund, Jonas W. Wastesson, Neda Agahi, Mårten Lagergren, Marti G. Parker

**Affiliations:** 1 Aging Research Center, Karolinska Institutet and Stockholm University, Gävlegatan 16, 113 30 Stockholm, Sweden; 2 Stockholm Gerontology Research Center, Stockholm, Sweden

**Keywords:** Life expectancy, Gender differences, Longevity, Old age, Gender gap

## Abstract

The female advantage in life expectancy (LE) is found worldwide, despite differences in living conditions, the status of women and other factors. However, this advantage has decreased in recent years in low-mortality countries. Few researchers have looked at the gender gap in LE in old age (age 65) in a longer historical perspective. Have women always had an advantage in LE at old age and do different countries share the same trends? Life expectancy data for 17 countries were assessed from Human Mortality Database from 1751 to 2007. Since most of the changes in LE taking place today are driven by reductions of old age mortality the gender difference in LE was calculated at age 65. Most low-mortality countries show the same historical trend, a rise and fall of women’s advantage in LE at age 65. Three phases that all but two countries passed through were discerned. After a long phase with a female advantage in LE at 65 of <1 year, the gender gap increased significantly during the twentieth century. The increase occurred in all countries but at different time points. Some countries such as England and France had an early rise in female advantage (1900–1919), while it occurred 50 years later in Sweden, Norway and in the Netherlands. The rise was followed by a more simultaneous fall in female advantage in the studied countries towards the end of the century, with exceptions of Japan and Spain. The different timing regarding the increase of women’s advantage indicates that country-specific factors may have driven the rise in female advantage, while factors shared by all countries may underlie the simultaneous fall. More comprehensive, multi-disciplinary study of the evolution of the gender gap in old age could provide new hypotheses concerning the determinants of gendered differences in mortality.

## Introduction

During the last 160 years, the record life expectancy (LE) at birth has increased at a steady pace (Oeppen and Vaupel 2002). Most gains in LE during this time have been achieved by reducing mortality at younger ages. It is only in the last five decades that reductions in old age mortality have had an impact on life expectancy at birth (Wilmoth et al. 2000; Oeppen and Vaupel 2002). During the same time the gender differences in life expectancy at birth grew for the most part of the twentieth century, but started to decline around the 1980s in Western countries (Oksuzyan et al. 2010).

Although there is remarkable variation in LE between different parts of the world, two similarities are shared: (1) both women and men show general increases in LE in most countries (Leon 2011), and (2) women outlive men in all countries (Barford et al. 2006). The female advantage is found worldwide, despite differences in living conditions, the status of women and other factors. In most low-mortality countries, the female advantage in life expectancy has been narrowing since the 1970s (Meslé 2004). Possible explanations for the decreasing gender difference in LE have been suggested and discussed in many countries; most frequently highlighting the changing smoking patterns among men and women (see e.g. Nilsson and Simonsson 2009). Discussions have commonly been country specific, few attempts for international comparisons have been made (Waldron 1993; Trovato and Heyen 2006; Glei and Horiuchi 2007; Meslé 2004). Since vital statistics are collected nationally, most researchers have focused on domestic-based differences and few researchers have studied the gender gap over a longer period of time.

A female advantage in longevity is also widespread among animals (Austad 2006), although it is far from universal (Austad 2011). Sex differences in LE can arise as a result of biological (intrinsic) factors, such as genetic and physiological advantages. They can also arise from contextual (extrinsic) factors, such as environmental and behavioural factors (Kirkwood and Austad 2000; Gems and Riddle 2000; McCulloch and Gems 2003); often to males’ disadvantage (Bonduriansky et al. 2008). Extrinsic and intrinsic factors are likely to interact in their influence on LE.

Most historical analyses of LE are based on life expectancy at birth and less is known about the development of the gender gap in old age mortality over time. The focus of this study is the gender gap in life expectancy at age 65. Focusing on remaining LE at 65 minimises some of the extrinsic factors related to mortality in younger ages, such as differential infant mortality risks, obstetrical mortality among women, and risk-taking behaviour and exposure to hazardous working conditions among men. Using LE at 65 also reduces the impact of different national figures regarding high alcohol consumption and smoking among younger men (Nusselder et al. 2010).

This paper focuses on the gender gap in LE at age 65 in a historical and global perspective. The first objective of this study is to describe gender differences in human longevity using vital statistics from different countries over an extended period of time. Sweden is the country with the oldest vital statistics in the world (starting in 1751). Thus, we begin by presenting the development of life expectancy for women and men at age 65 in Sweden from 1751 until 2007. Then, gender differences in LE for 17 countries with data prior to 1950 are given. By focusing on similarities among countries, our aim is to display a more complete picture of how gender differences in life expectancy have developed in a historical and global perspective. Have women always had an advantage in LE? Have there been changes over time in the LE gender gap? Have these changes been similar in all countries? Using extended time series, we can identify historical fluctuations in the gender gap in the different countries to search for similarities and differences in the fluctuations over time.

The second objective of this study is to discuss these LE fluctuations in relation to historical trends in other gender-related factors (given lag effects) such as smoking, access to education, warfare mortality and working conditions. In light of what is known about the cultural, social and biological factors related to sex differences in aging, gender gap trends in LE can contribute to the generation of hypotheses about underlying gender-related LE determinants.

## Materials and methods

The Human Mortality Database (HMD) (Human Mortality Database Date accessed: February 10, 2010) is a collaborative project, launched in 2002, involving research teams at the Department of Demography at the University of California, Berkeley (USA) and at the Max Planck Institute for Demographic Research (MPIDR) in Rostock (Germany). It contains death rates and life tables for national populations (countries or areas), as well as the input data used in constructing those tables. The input data consist of death counts from vital statistics, plus census counts, birth counts, and population estimates from various sources. The data for LE 65 is calculated in a comparable way for all included countries. The data quality is generally good, however, data collected prior to 1950 can be affected by methodological issues and should be regarded with higher caution.

All countries with data prior to 1950 and without methodological issues (after 1950) according to HMD, were selected for this study. Iceland was excluded since data fluctuated considerably due to its small population. Since only countries with data prior to 1950 were included in the sample, the included countries were those with a long tradition of keeping birth and death counts. These countries are today generally countries of relative affluence and low-mortality (LE at 65 ranges from 84 to 88 years for women and 81 to 83 years for men in 2000–2007). Seventeen countries were included in the study: Sweden (SWE), United States (US), England and Wales (ENG and W), France (FRA), Japan (JPN), Finland (FIN), Denmark (DNK), Italy (ITA), Norway (NOR), the Netherlands (NLD), Canada (CAN), Belgium (BEL), Switzerland (CHE), Australia (AUS), Spain (ESP), New Zealand (NZL) and Austria (AUT). The earliest available statistics are from 1751 (Sweden) and the latest from 1948 (New Zealand).

Life expectancy is an estimate of the number of years a person can expect to live under the mortality conditions of that specific year. We report the gender differences in LE at 65 (delta LE at 65) in absolute numbers (actual years) since we do not want to give larger impact to smaller numbers. For example, a 1-year difference would have looked larger (in ratios) in the beginning of the study period, when life expectancy was lower than in more recent years when life expectancy has increased.

## Results

**Fig. 1:**
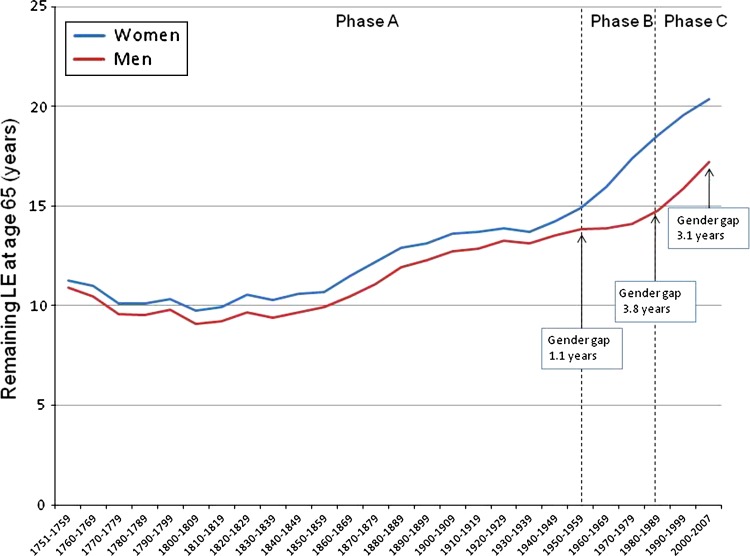
Remaining life expectancy (LE) at age 65 for men and women in Sweden, 1751–2007

Figure 1 presents remaining life expectancy at age 65 for men and women in Sweden, from 1751 until 2007. The graph shows that from the mid eighteenth century and into the nineteenth, women had a remaining LE at 65 of about 11 years and men about 10 years. In the 1840s, LE began to increase for both men and women and has continued to rise ever since. In 2007, LE at 65 was 20 years for women and 17 years for men.

Focusing on the gender differences in LE in Fig. 1, three phases can be discerned. During 200 years, women’s advantage was stable at <1 year (Phase A). In the 1950s, the gender gap began to increase beyond the 1-year gap (Phase B), mainly due to LE improvements among women. In the 1970s, the gap began to diminish (Phase C) as men’s survival improved.

**Fig. 2:**
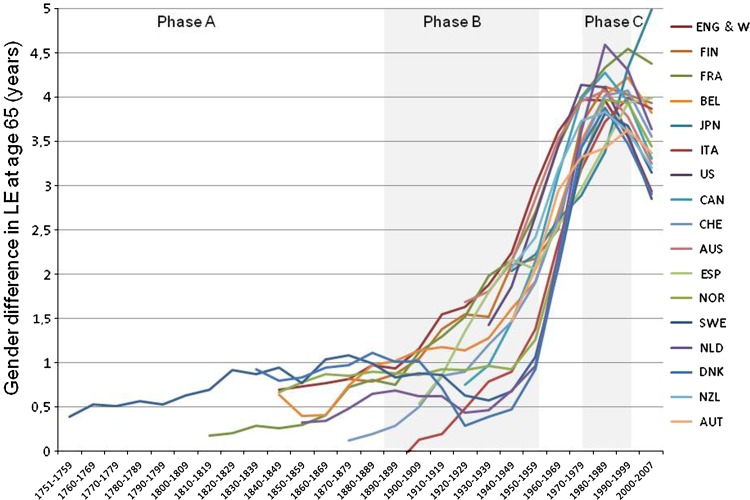
Gender difference in life expectancy (LE) at age 65 (in years) in 17 countries, 1751–2007

Figure 2 depicts the gender differences in LE at age 65 in different countries during the last centuries. As in Sweden, all included countries show a progression from a period with a slight advantage for women (Phase A), to a rapid increase in women’s advantage (Phase B), followed by a decrease in the gender gap (Phase C). All countries seem to pass through all phases, with the exceptions of Japan and Spain that did not show a decrease (Phase C). The years of entrance into Phases B and C differ between countries (the shaded areas in Fig. 2).

Phase A is characterized by a women’s advantage of <1 year. During this period, chances of surviving to advanced ages were low. In Sweden, for example, the probability of surviving to age 65 was 23 % among men and 29 % among women in 1751, and 49 and 54 %, respectively, around 1900.

During Phase B, women’s advantage in LE increased. From a stable difference of about 1 year, LE for men and women diverges in all represented countries and reaches a discrepancy of about 4 years around the 1980s. However, the onset of the rise varies considerably between the countries, as seen in the shaded area in Phase B in Fig. 2. The first countries to enter Phase B are England, France and Finland around the turn of the twentieth century, whereas Sweden and the Netherlands enter more than 50 years later. In countries with later debuts, women’s advantage increased at a faster rate, so that all countries reached a 4-year gender gap at about the same time.

Phase C is distinguished by another trend shift: the rise in gender differences ends abruptly and within a period of 20 years (approximately 1970–1990), women’s advantage in longevity decreases in all included countries except Japan and Spain. The curve for Spain levels off, while the advantage for Japanese women continues to rise.

The probability to survive to age 65 continued to increase during Phases B and C. In Sweden, 62 % of men and 67 % of women survived from birth to age 65 in the year 1930–1939. In 1980–1989, the rates were 79 and 89 %, respectively.

## Discussion

The pattern of change in gender differences in LE at 65 is remarkably similar over time in the studied low-mortality countries. Three phases could be identified in all countries, with two exceptions. Until the twentieth century, the included countries had a gender difference of 1 year or less in favour of women (Phase A). This was followed by a period with differences increasing to about 4 years (Phase B). In most countries, the peak was seen during the 1970–1980s. Since the 1980s, the gender difference in LE at 65 has been decreasing in all of the studied countries (Phase C) except Japan and Spain. However, recent data suggests that the gender gap in Japan has also begun to decrease (Liu et al. 2012a).

We find it noteworthy that most of the countries, that are considered low-mortality countries today, share a common development in LE, although historically they have had different experiences of war, urbanisation, welfare systems, industrialisation and migration. Countries also show similar patterns of LE gender differences, despite differences in gender roles and opportunities for women.

Furthermore, it is intriguing that although most countries share the same trends, the timing of the phases differs between the countries. The first trend shift—the increase of the gender difference—occurred over a period of 50 years starting with England and France. The shift came much later in Sweden, Norway and the Netherlands. On the other hand, the more recent narrowing of gender differences happened over a much shorter time.

Studying this inter-country variability—and similarity—can help to identify possible mechanisms driving the changes in the LE gender gap. These long-term trends in LE can be put in a historical context. In the eighteenth and nineteenth centuries, mortality rates were high due to extrinsic factors, such as epidemics and adverse living conditions. Less than half of the population survived to 65 years of age. Historically, this was a period of relatively low economic growth in many of the countries. Women’s advantage in LE was stable over time at about 1 year (Phase A).

The twentieth century was characterised by exceptional economic growth, industrialisation and urbanisation as well as medical advances in many countries. Attempts to explain the widening of the longevity gender gap (Phase B) have primarily been nationally based and have focused on environmental and societal factors. Industrialisation and urbanisation have been suggested to be more favourable for women, e.g. due to men’s adverse working conditions and poorer health habits (Mooney 2002). Women’s suffrage, entrance into the labour market, and safer child bearing also favoured women (Nobles et al. 2010; Pampel and Zimmer 1989). Although these factors primarily influence mortality in younger ages, they also affect the chance to reach old age as well as longevity in old age.

An alternative interpretation is that the widening gender gap in Phase B is an indication of a biological female longevity advantage. If mortality is largely driven by social factors at younger ages, does biology take a larger toll at old age? From an evolutionary perspective, extrinsic factors are the most important determinants of survival in a population. In a society with a high level of extrinsic hazards, distributed equally, women and men will have a low and similar LE (as in Phase A). Those who escape the hazards of extrinsic mortality face the effects of the aging process, i.e. cellular, DNA, tissue and organ damage which the body is unable to repair (Kirkwood and Austad 2000; Kirkwood 2005), ultimately leading to intrinsic (or age-related) mortality. As extrinsic hazards level off, intrinsic rather than extrinsic mortality begins to take a toll, possibly revealing a biological longevity advantage of women. An array of hypotheses has been proposed to explain why females could have an advantage, e.g. oestrogen levels, oxidative stress and reproduction (Oksuzyan et al. 2008; Kirkwood 2001).

However, if increases in LE were to unmask a biological advantage in women, countries with an increasing LE should also show an increasing gender gap as extrinsic mortality decreases and more people reach 65. Demographic data, however, do not support this. While probabilities to survive to age 65 increased remarkably during the early twentieth century, a higher survival to age 65 did not necessarily prelude increasing gender differences in LE. On the contrary, the first countries to show rising gender differences had lower probabilities of reaching 65 than the late risers. In 1900, when the gender gap began to rise in France, England and Finland, chances of surviving to age 65 ranged between 36–38 % for men and 43–46 % for women; while the Netherlands, Sweden and Norway had probabilities of 46–49 % for men and 50–54 % for women to survive to age 65 in the same year. In 1950, similar inter-country differences in the probability of surviving to age 65 remained. Therefore, a biological female advantage cannot be the only explanation for the increase in the LE gender gap. Nor can it explain the more recent decrease in the gender gap.

The increasing gender difference came to a remarkably simultaneous halt between 1970 and 1990 in almost all studied countries, followed by a decrease (Phase C) that is still ongoing (Waldron 1993; Trovato and Heyen 2006; Glei and Horiuchi 2007). While entrance into Phase B showed wide variations between countries, entrance into Phase C occurred during a narrower time margin. This period is characterised by further improvements in living conditions and continued increases in LE driven mainly by reduced mortality in old age (Vaupel 2010; Wilmoth et al. 2000). Also, from the 1970s and onward the timing of demographic events, such as mortality trends, seems to have become more similar across countries (Leon 2011). However, two countries have not yet entered Phase C, Japan and Spain. One reason suggested for the increasing gender inequality in Japan is respiratory diseases. At very high ages, Japanese women’s rate of respiratory disease is stable whereas it is increasing among men (Meslé 2004).

In most countries, the reduced gap is related to increased LE gains among men rather than losses among women (Meslé 2004; Meinow et al. 2010). A cross-national comparison showed that the narrowing of the female advantage was mainly due to reductions in cardiovascular mortality among men in the Nordic countries, England and Wales, whereas the narrowing of the gap in the Mediterranean countries was mainly due to reductions in male cancer mortality (Meslé 2004). Reductions in cardiovascular mortality are often attributed to both medical advances and improved lifestyle habits (Rosén and Haglund 2005). The medical and technical advances, especially those relating to cardiovascular health and mortality, may have favoured survival among older men to a greater extent than older women (Rosén and Haglund 2005). Also, lifestyle habits have gradually become more similar among men and women during this period, e.g. men have quit smoking to a large extent while women have taken up the habit (Lopez et al. 1994).

In Sweden, as elsewhere, a common hypothesis has been that the changes in smoking habits over time—initially a male habit gradually becoming a female habit, explain both the widening (Phase B) and the narrowing of the gap (Phase C). Although smoking probably has played an important explanatory role for the changes in gender difference in LE in some of the studied countries (Meslé and Vallin 2006), smoking does not account for the total gender difference in LE (McCartney et al. 2011). Further, from a cross-national perspective, the gap emerged too early in some countries (England, France and Finland) to be a consequence of increased male smoking habits (Ravenholt 1990).

Many questions remain. Will LE continue to rise for both women and men during the coming centuries? The prediction that 50 % of all children born in England and the US in the beginning of this millennium will live to be a 100 years old (Christensen et al. 2009) rests on a number of assumptions (e.g. unchanged mortality rates before age 50 and continued improvements in mortality rates in old age) that have been questioned by others (Olshansky and Carnes 2010). Will the gender gap in LE continue to decrease? According to our own calculations, the longevity gender gap in Sweden will be eliminated by the middle of this century, if the current rate of decrease continues (Lagergren; calculations not shown) or will the gap increase once again as smoking-related mortality levels off among women, allowing the hypothesised female biological advantage to re-emerge (Pampel 2002)?

This paper focuses on general similarities between countries in the development of sex differences in LE at age 65. Many aspects of life expectancy remain to be further investigated, e.g. probabilities to survive to 65 years. When investigating the mechanisms behind life expectancy in old age it is important to take into account that the increasing proportion of people surviving to old age in a population, most likely leads to a weaker selective survival. More study of selective survival, and how this can affect gender differences in LE, are called for.

Another factor to consider is the fact that men have had greater variation in age at death compared to women. Over time, men’s deaths have compressed to a narrower age range and their survivorship has become more rectangular, similar to women’s. Glei and Horiuchi (2007) explain that this could have contributed to the decreased gender gap in LE (Phase C). However, it does not fully account for the reduction of the gender gap.

The study of populations over time is complex. Life expectancy differences should ideally be studied in relation to country-level differences. The risk of “ecological fallacy,” whereby inferences about individuals are based on aggregate data, is obvious in the study of population-level determinants of gender differences. However, studying gender differences across countries in relation to macro-level structural indicators could be a useful method for understanding and generating new hypotheses about gender differences in old age. For example, in the European Union, Van Oyen et al. (2010) have shown that gender differences in activity limitations are affected by macro-level indicators such as: gross domestic product, expenditure on elder care and income inequality in recent years. An analysis of OECD countries showed a negative association between the gender gap in LE and country-specific social development indices (Liu et al. 2012b). Further research is needed to understand how these factors have influenced the gender gap in LE and the transitions between Phases A, B and C. From a historical perspective, many factors seem well worth studying in relation to gender differences in mortality: economic growth, urbanisation and industrialisation, the experience of war and the expansion of welfare institutions such as public schools, pension systems and health care systems.

Vital statistics provide the ‘big picture’ that can challenge assumptions and generate hypotheses about how political, social and economic change, along with health behaviours such as smoking, alcohol consumption and obesity, contribute to changes in LE (Leon 2011). Likewise, they reflect how changes in living conditions and social roles can affect men and women differently, as seen in the gender gap in LE. The concept of the three phases of the LE gender gap outlined here can be used as a framework on which further work can be based. By comparing trends in, e.g. gender differentiated behaviour or living conditions, against the trends in the LE gender gap in the different countries, we may be able to identify factors that impact LE differently for men and women. For example, the timing of change in smoking behaviour among women and men was different in different countries. Is this timing (accounting for a possible lag effect) associated with entrance into Phase B (the increase in LE gender gap)? Of particular interest would be to investigate factors associated with entrance into Phase C—a transition that occurs almost simultaneously in all countries except two. Do the latter factors represent some aspect of globalisation among low-mortality countries? Which of these factors are potentially modifiable?

Investigating gender differences in LE can ultimately advance understanding of the intertwined and synergistic processes determining longevity. Despite decades of research, the role of behavioural, biological and social factors in gender differences in LE remain confusing if not elusive. Change in the LE gender gap cannot be understood in the context of a single country or a single discipline. The multifaceted nature of demographic and socioeconomic change calls for a broad multidisciplinary approach when analysing differences (and similarities) between countries and populations over time. Demographers and epidemiologists need to collaborate with historians and social scientists as well as with biologists and medical scientists if we are to understand the forces driving longevity.

In conclusion, our findings indicate that the studied countries have shown similar patterns in the LE gender gap at age 65, but with different timing. However, the timing of gender gap change seems to have become more similar over time, which could indicate that the factors driving LE gender differences in old age have become increasingly international. More international and historical investigation of the trends identified in this study could provide new hypotheses for research and better understanding of the gender gap in old age mortality.
